# Does a historical catch record support a stable latent structure in catch composition across ports, taxa, and years? An assessment of cooperative-level catch statistics from Lake Biwa, Japan, 1962–1972

**DOI:** 10.1371/journal.pone.0358946

**Published:** 2026-10-14

**Authors:** Noriaki Kondo, Tamaki Kashio, Kenichi Satoh, Kunihiko Matsumoto

**Affiliations:** 1 Data Science and AI Innovation Research Promotion Center, Shiga University, Hikone, Shiga, Japan; 2 Lake Biwa Museum, Kusatsu, Shiga, Japan; 3 Faculty of Data Science, Shiga University, Hikone, Shiga, Japan; 4 College of Science and Engineering, Kanazawa University, Kanazawa, Ishikawa, Japan; National Cheng Kung University, TAIWAN

## Abstract

Historical inland-fisheries records resolved by taxon, location, and year are rare, and whether such records support stable low-dimensional structure is an empirical question. We analyzed annual catch records from Lake Biwa, Japan, covering 44 fisheries cooperatives, 13 taxonomic categories, and 11 years (1962–1972), immediately preceding major coastal modifications associated with the Lake Biwa Comprehensive Development Project. We assessed whether non-negative matrix factorization (NMF), with and without spatial smoothing based on port coordinates, provided a stable representation of port-year catch composition beyond direct summaries. Candidate ranks *Q* = 1–8 were evaluated using bi-cross-validation, 30 random starts, consensus diagnostics, port-block bootstrap resampling, exclusion analyses, and sensitivity analyses. Bi-cross-validation reached its minimum at *Q* = 8, with the one-standard-error rule selecting *Q* = 7, whereas component correspondence across random starts deteriorated sharply at *Q* ≥ 6. Because these criteria did not agree, *Q* = 4 was used as an operational reference under a study-specific stability rule rather than as a uniquely selected optimum. At *Q* = 4, three components dominated by Crucian carp, Freshwater clam, and Landlocked Ayu were reproducible and closely tracked the corresponding observed shares, whereas a mixed fourth component was less stable. Repeating the stability analyses at *Q* = 5 produced the same qualitative pattern of a reproducible core and less stable additional structure. Component correspondence also depended on data representation. Some observed shares and component scores showed spatial autocorrelation among reporting ports, whereas reconstruction error did not. NMF therefore identified a reproducible core representation of recorded catch composition without supporting a unique and uniformly stable latent structure. These patterns describe recorded catch composition rather than stock abundance and provide a historical reference for future comparison.

## 1 Introduction

The assessment and management of multispecies fisheries require species within a given system to be considered jointly, while model complexity must remain consistent with the limitations of the available data [1–3]. In marine fisheries, where multispecies analyses are more extensively developed, such analyses typically rely on records that identify catch and, where available, fishing effort by species, location, and time. Catch-and-effort standardization similarly depends on observations resolved by species, location, and time [4].

Inland fisheries, by contrast, seldom provide records of comparable detail. Global inland catch statistics are typically compiled as national or broad-area aggregates and are susceptible to underreporting and inconsistencies in reporting methods [5–8]. In some cases, incomplete observations have required expert knowledge to supplement conventional statistical data [7]. Subsistence catches and fish consumed within fishing households may also be underreported in official statistics [8]. In Japan, inland fishery statistics are typically aggregated by prefecture, water body, and taxonomic category [9]. Guidance on inland fisheries data collection consistently emphasizes the importance of fishing-effort information and landing-site resolution [10–12], yet historical inland fisheries statistics rarely contain both. Whether a given historical record supports analysis of catch composition across taxonomic categories, locations, and years is therefore an empirical question.

The present study examines cooperative-level catch tables for Lake Biwa published in Fisheries of Lake Biwa [13]. Although the title of the source volume refers to 1960–1972, the cooperative-by-category tables it contains cover 1962–1972 only; this 11-year period defines the record analyzed here. Lake Biwa, the largest freshwater lake in Japan, supports long-standing commercial fisheries and a diverse freshwater fauna, including endemic species [14,15]. Among the sources reviewed for this study, this is the only published record identified in which catches are reported jointly by fisheries cooperative and taxonomic category around the lake. More recent official statistical series use different levels of aggregation and do not preserve the same cooperative-level panel structure [9]. The analytical dataset is a balanced panel comprising 44 fisheries cooperatives, 11 years, and 13 taxonomic categories retained for analysis. Each cooperative is associated with a landing port; throughout this study, these locations are treated as landing and reporting points rather than as fishing grounds.

The 1962–1972 record ends in the same year as the enactment of the Lake Biwa Comprehensive Development Special Measures Act. It can therefore serve as a historical reference for future comparisons; however, the present study does not estimate the effects of the Lake Biwa Comprehensive Development Project. Moreover, the absence of data on fishing effort and fishing grounds precludes direct interpretation of the recorded catches as measures of stock abundance, species distributions, or habitat use. The scope of inference is therefore limited to patterns in recorded catch composition and their variation among reporting units and years.

Previous research on Lake Biwa fisheries has documented regional differences in fishing grounds, fishing gear, and catch composition [16] and has examined lake- or basin-wide catch trends and their environmental correlates [17,18]. Other studies have investigated the marketing, processing, culinary use, and cultural significance of lake fish [19,20], as well as the role of fishers in lakeshore conservation [21]. The studies most closely related to the present analysis [17,18] examine catch series aggregated by lake or basin and organized by taxonomic category. Elsewhere, historical catch records have been recovered and analyzed at fine spatial scales, for example, spatially resolved commercial landings off California [22] and century-long catch records for a single Caribbean island [23]. Multivariate methods such as principal component analysis and clustering are well established for identifying fishing tactics and métiers from multispecies catch compositions [24,25].

However, they do not assess whether cooperative-level historical records support stable and interpretable latent patterns in recorded catch composition across taxonomic categories, landing locations, and years. More broadly, historical information has been advocated as a means of addressing the progressive loss of fisheries reference points [26], and historical inland catches have been reconstructed or allocated at broader geographical scales [27]. What remains unresolved is whether a rare, spatially disaggregated historical inland-fisheries record supports a stable and interpretable latent structure at the scale at which catches were originally reported.

This study therefore asks whether the 1962–1972 Lake Biwa cooperative-level catch record supports stable and interpretable latent patterns in recorded catch composition across associated landing ports, taxonomic categories, and years. Here, latent patterns denote a small number of taxonomic composition profiles shared across the record, together with the weight of each profile in every port-year. We first characterize the record using direct summaries of catch magnitude, composition, concentration, dominance, and spatial and temporal variation. We then apply non-negative matrix factorization (NMF) and evaluate the resulting structure through analyses of model rank, initialization, resampling, exclusion analyses, preprocessing, alternative treatments of recorded zeros, and spatial-kernel specifications. NMF is used as an established analytical tool; no methodological novelty is claimed. We directly compare NMF component scores with observed taxonomic shares to assess whether the latent representation provides information beyond marginal summaries. Accordingly, this study evaluates the stability and interpretability of catch-composition patterns supported by this historical record rather than the record’s general adequacy for stock assessment or fisheries management. The outcome of this assessment provides a reference for judging whether comparable historical records can support analyses of this kind.

## 2 Materials and methods

### 2.1 Study area

Lake Biwa is the largest freshwater lake in Japan, located in Shiga Prefecture, central Honshu (Fig 1). The lake has a surface area of 669.26 km^2^, a water volume of 27.5 km^3^, and a shoreline length of approximately 235 km. It is fed by more than 460 rivers, and the Seta River is its only natural outlet [14,15]. The lake extends approximately 60 km from north to south.

**Fig 1:**
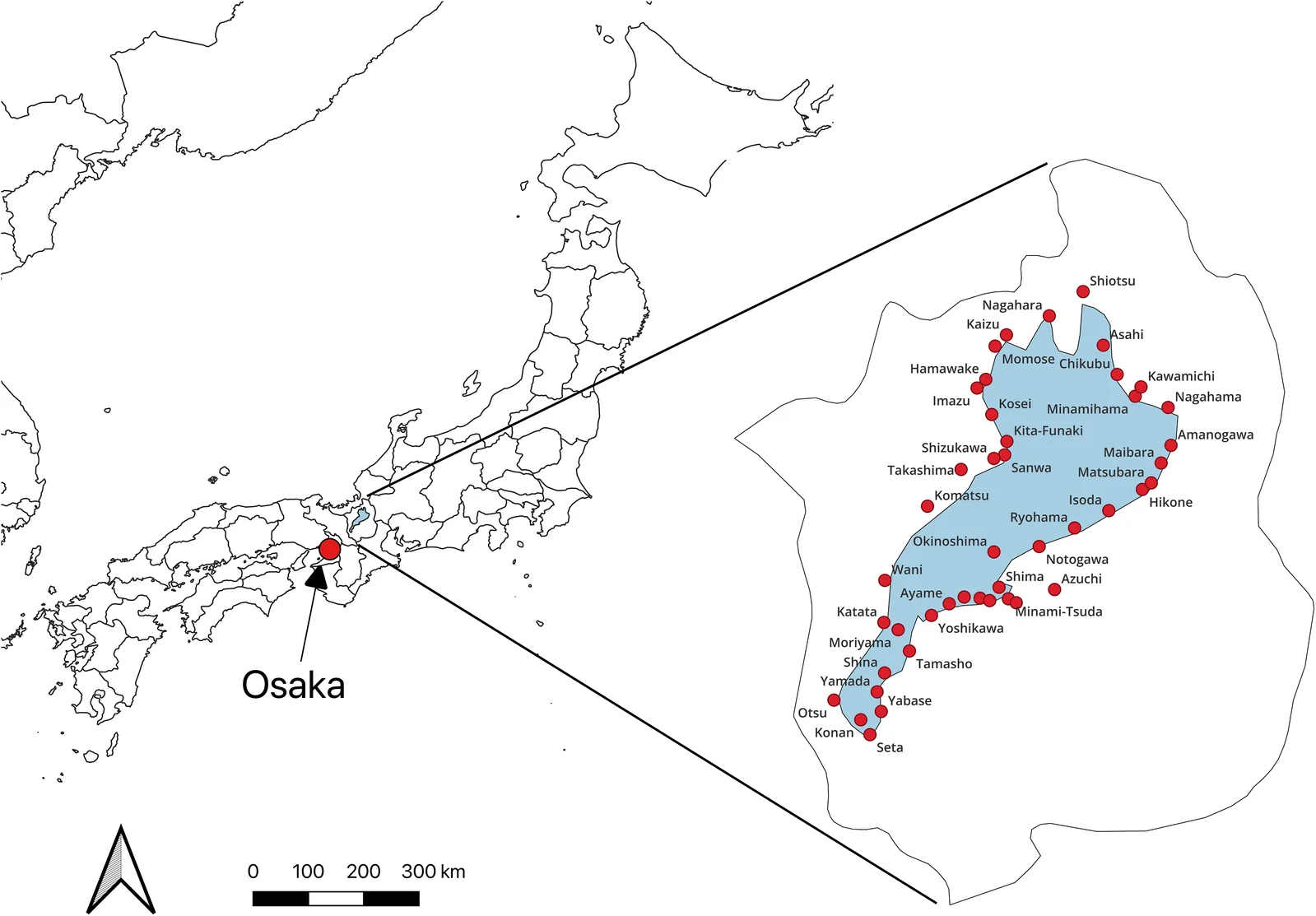
Location of Lake Biwa and the 44 ports included in the analysis. The map shows Lake Biwa within Shiga Prefecture and its location in Japan, and the 44 ports associated with the fisheries cooperatives in the historical catch record. The map was created by the authors in QGIS using public-domain vector data from Natural Earth. The locations of the 44 ports were plotted using coordinates identified from official fisheries maps and records published by Shiga Prefecture [28] and deposited in the reproducibility archive. Fig 1 uses WGS84 geographic coordinates (EPSG:4326). No proprietary basemap or satellite imagery was used.

Lake Biwa comprises two distinct limnological basins. The northern basin is substantially larger and deeper, with a maximum depth of 104 m, a mean depth of 44 m, and a volume of approximately 27.3 km^3^. The southern basin is smaller and shallower, with a maximum depth of 8 m, a mean depth of 3.5 m, and a volume of approximately 0.2 km^3^ [14,15]. The two basins are connected at the narrow section near Katata. In this study, the terms “northern basin” and “southern basin” refer exclusively to these physical divisions of the lake; they are not used as analytical units in the NMF decomposition.

The fisheries cooperatives documented in the historical catch record are associated with landing ports distributed around Lake Biwa. The geographic coordinates of these ports indicate where catches were landed and reported. They are not interpreted as fishing grounds, species habitats, or locations of stock abundance. The role of spatial proximity among ports is evaluated explicitly in the spatial sensitivity analyses described below.

### 2.2 Data

Catch data were transcribed from Fisheries of Lake Biwa, published in 1973 by the Kinki Regional Agricultural Administration Office and the Shiga Statistical Information Office [13]. Although the title of the source volume refers to 1960–1972, the cooperative-by-category tables it contains cover 1962–1972 only. The analytical panel therefore comprises 44 fisheries cooperatives observed annually over 11 years, yielding 484 cooperative-year observations. All 44 cooperatives are represented in every year.

Each cooperative in the historical source is identified by the name of its base port. The locations of the 44 ports were identified from official fisheries maps and records published by Shiga Prefecture [28]. Port coordinates were recorded in decimal degrees (WGS84, EPSG:4326). For spatial analyses, the coordinates were projected to JGD2011 / Japan Plane Rectangular CS VI (EPSG:6674), and pairwise Euclidean distances were calculated in kilometers. The median nearest-neighbor distance among ports was 2.37 km, and the maximum pairwise distance was 63.8 km. Cooperative-year observations are identified by their associated port names in the dataset and figures. The coordinates represent landing and reporting locations and are not interpreted as fishing grounds, species habitats, or locations of stock abundance.

13 taxonomic categories (hereafter, “categories”) were retained for analysis. The category labels used throughout the analyses, figures, and deposited dataset are identical and are listed in Table 1. These are historical catch categories and do not necessarily correspond one-to-one with currently recognized species. Two categories are composite. Trout represents the historical masu category, which encompasses multiple salmonid taxa. Willow gudgeon combines honmoroko with the separate source category “other moroko”; this combination was made during transcription and is represented by a single Willow gudgeon column in the deposited dataset. Present-day taxonomic correspondence was based primarily on Kawanabe et al. [15], with specific nomenclatural revisions supplemented by the historical and taxonomic sources described below.

**Table 1:** Analyzed taxonomic categories and their present-day taxonomic correspondence.

Analyzed category, deposited column, and figure label	Present-day taxonomic correspondence	Source category (romanized)
Trout^a^	*Oncorhynchus* spp.	masu
Landlocked Ayu	*Plecoglossus altivelis altivelis*	koayu
Common carp	*Cyprinus carpio*	koi
Crucian carp	*Carassius* spp.	funa
Japanese dace	*Pseudaspius hakonensis*	ugui
Eel	*Anguilla japonica*	unagi
Isaza goby	*Gymnogobius isaza*	isaza
Willow gudgeon^a^	*Gnathopogon caerulescens* and other moroko taxa	honmoroko & other moroko
Three-lips	*Opsariichthys uncirostris*	hasu
Oriental river prawn	*Macrobrachium nipponense*	tenagaebi
Lake prawn	*Palaemon paucidens*	sujiebi
Freshwater clam	*Corbicula sandai*	shijimi
Pearl mussel	*Hyriopsis schlegelii*	bokai

Category labels are those of the deposited dataset and are used unchanged in all analyses, tables, and figures. Present-day names are reference labels only and do not redefine the historical categories.^a^ Composite historical category (see Methods).

Taxonomic nomenclature has changed since the historical source was compiled, and several historical categories cannot be assigned one-to-one to a single currently recognized species. Kuroda [29], for example, used *Gnathopogon elongatus caerulescens* and *Chaenogobius annularis isaza* for taxa corresponding to categories now represented as *Gnathopogon caerulescens* and *Gymnogobius isaza*. The placement of Japanese dace in *Pseudaspius* follows the revised generic taxonomy of Sakai et al. [30]. The scientific names in Table 1 are therefore used only as present-day taxonomic reference labels; all numerical analyses use the historical catch categories listed in the first column.

The analytical matrix excludes identifiers and preparation metadata that contain no catch information. The transcription also includes “other fish” and “other shellfish.” These categories were retained for data-quality checks but excluded from the NMF analysis because they cannot be assigned consistently to the 13 analyzed categories. The printed Total was also excluded from construction of the compositional matrix and was used only for internal-consistency checks. All compositional denominators were calculated as the sum of the 13 analyzed categories. After the verified corrections documented in S1 Text and S1 Table, the 13-category analytical matrix contains a total recorded catch weight of 58,241,377 kg.

The historical catch data were transcribed and checked for internal consistency before analysis. For each cooperative-year, the printed Total in the source was compared with the sum of the 15 transcribed detail categories, comprising the 13 categories used in the analysis plus “other fish” and “other shellfish.” Because the printed Total includes items not represented among these 15 categories, a Total greater than the transcribed detail sum does not in itself indicate a transcription error; a detail sum exceeding the Total, or a large shortfall, may.

Using the uncorrected transcription, cooperative-years were flagged when the absolute difference between the printed Total and the transcribed detail sum was at least 5,000 kg or at least 25% of the printed Total. The 45 cooperative-years so identified were checked against the printed source, and transcription errors found in this way were corrected to the printed values (S1 Table). For the 27 cooperative-years whose discrepancies could not be resolved, the transcribed values were retained unchanged because the historical record provides no basis for determining which figure is correct (S2 Table). The verification criteria and the unresolved cases are documented in S1 Text and S1–S2 Tables.

The 13-category analytical matrix contains 6,292 cells, of which 2,131 (33.9%) are recorded as zero. These entries were retained as reported values rather than treated as missing data. However, the historical source does not allow a recorded zero to be uniquely attributed to zero catch, zero fishing effort, the absence of relevant fishing gear, market or reporting practices, or other processes. Sensitivity to the treatment of recorded zeros was therefore evaluated explicitly in the analyses described below. No cooperative-year observation is zero across all 13 analyzed categories.

Catch quantities were analyzed as recorded weights. For the Freshwater clam category (shijimi; *Corbicula sandai*), the reported weight is shell-on. This category accounts for 46.0% of the total recorded weight in the corrected 13-category matrix. Its contribution to catch composition therefore represents shell-on recorded catch weight and should not be interpreted as soft-tissue biomass, individual abundance, or stock abundance. More generally, because the historical record contains no fishing-effort data, recorded catch weights are treated as catch records rather than as direct measures of biological abundance.

The machine-readable transcription, port coordinates, correction and verification ledgers, analysis code, the exact source of the nmfkc package used in the analyses, fitted objects, and derived outputs necessary to reproduce the findings are publicly available in Zenodo (version https://doi.org/10.5281/zenodo.22336084).

### 2.3 Analytical methods

#### 2.3.1 Analytical framework.

The analysis examined whether the historical record supports stable and interpretable patterns in catch composition across taxonomic categories, ports, and years. The 484 cooperative-year observations, identified by their associated port names, are hereafter referred to as port-years. Let *y*_ikt_ denote the recorded catch weight of category *i* at port *k* in year *t*, and let


$$\begin{array}{c} T_{kt}=\sum_{i^{\prime }=\text{1}}^{\text{1}\text{3}}y_{i^{\prime }kt}\text{.} \end{array}$$ (1)

Catch composition was defined as


$$\begin{array}{c} p_{ikt}=\frac{y_{ikt}}{T_{kt}}\text{.} \end{array}$$ (2)

The resulting matrix *P* contained 13 taxonomic categories as rows and 484 port-years as columns, with each column summing to one. Columns were ordered by year and, within each year, alphabetically by port. The printed Total, “other fish,” and “other shellfish” were not used in constructing *P*.

Before applying NMF, the record was characterized directly using recorded catch weight; taxonomic shares; the maximum share and the category holding it (the dominant category) in each port-year; compositional concentration within each port-year, measured by the Herfindahl–Hirschman index (HHI) of the 13 shares; the concentration of each category’s recorded weight among the 44 ports within each year, measured by the HHI of the category’s weight across ports; and recorded-zero frequency. Change in composition between consecutive years of the same port was summarized by the total-variation distance,


$$\begin{array}{c} TV_{k,t}=\frac{\text{1}}{\text{2}}\sum_{i=\text{1}}^{\text{1}\text{3}}\left|p_{ik,t}-p_{ik,t-\text{1}}\right|\text{.} \end{array}$$ (3)

These direct summaries provided the empirical basis for interpreting the NMF representation.

#### 2.3.2 Non-negative matrix factorization.

Non-negative matrix factorization (NMF) was used as an established dimension-reduction method following Lee and Seung [31,32]. The implementation used the covariate-extended formulation implemented in nmfkc version 0.9.4 [33]. At rank *Q* (the number of components),


$$\begin{array}{c} P\approx XB\text{,} \end{array}$$ (4)

where *X* is a 13 × *Q* matrix of non-negative taxonomic profiles and *B* is a *Q* × 484 matrix of component scores. The factors were estimated by minimizing $∥P-XB{∥}_{F}^{\text{2}}$ over non-negative *X* and *B* (or *C* in the spatial model, Section 2.3.3). Each taxonomic profile (column of *X*) was constrained to sum to one. Relative component scores were obtained by normalizing the fitted scores within each port-year so that they summed to one. These normalized scores were used in figures, Moran’s I and Spearman correlation analyses, and in the selection of exemplar port-years; unnormalized scores were used for component ordering and for the spatial-model score correlations. The primary model used squared Euclidean loss, equal cell weights, and no pseudo count or regularization penalty.

Models were estimated in R 4.4.0 using the multiplicative updates of Lee and Seung [31,32] as implemented in nmfkc [33]. Random-uniform initialization, a convergence tolerance of 10^−8^, and a maximum of 100,000 iterations were used; the tolerance and iteration limit are stricter than the package defaults of 10^−4^ and 5,000 iterations. Convergence was defined by


$$\begin{array}{c} \frac{\left|f_{j}-f_{j-\text{1}}\right|}{\text{max}\left(\left|f_{j}\right|,\text{1}\right)}\leq {\text{1}\text{0}}^{-\text{8}}\text{,} \end{array}$$ (5)

where $f_{j}$ is the objective value after iteration j. Each full-data specification was fitted from 30 random starts, seeded with the integers 1–30 (one seed per start). The fit with the smallest final objective value was retained as the representative solution, and all 30 fits were retained for stability assessment.

Reconstruction was summarized using squared Euclidean loss and the uncentered coefficient of determination, *R*_*u*_^*2*^, defined as


$$\begin{array}{c} R_{u}^{\text{2}}=\text{1}-\frac{{\left\|P-XB\right\|}_{F}^{\text{2}}}{{\left\|P\right\|}_{F}^{\text{2}}}\text{.} \end{array}$$ (6)

Because component labels are exchangeable across NMF fits, components were aligned by maximizing the sum of cosine similarities between matched taxonomic profiles over all component permutations. Components in the representative solution were then ordered by their total unnormalized score across port-years. The tagged nmfkc v0.9.4 source package used in the analysis (package date 14 August 2026) is included in the reproducibility archive [33].

#### 2.3.3 Validation and sensitivity analyses of NMF.

Candidate NMF ranks *Q* = 1–8 were evaluated using reconstruction performance, stability across random starts, consensus diagnostics, predictive validation, and effective rank. As a complementary data-level diagnostic, the effective rank of *P* was calculated from the entropy of its normalized singular values [34]. Stability across the 30 random starts was assessed using matched-profile cosine similarity and the range of final objective values. For *Q* = 2–8, consensus matrices were constructed from 30 random-initialization fits by assigning each port-year to its highest-scoring component and were summarized by the cophenetic correlation coefficient and the proportion of ambiguous clustering (PAC; ambiguous interval 0.1–0.9) [35–37]. Adjacent-rank persistence was assessed descriptively using the maximum cosine similarity between each component at rank *Q* and the components at rank *Q* + 1; this measure was not used as an inferential test or as a standalone rank-selection criterion.

The operational reference rank was defined by a study-specific stability criterion: all 30 starts had to converge, the minimum matched-profile cosine similarity after rounding to three decimal places had to equal 1.000, and PAC had to be ≤ 0.005. This criterion was introduced during revision, after examination of the rank diagnostics, as an operational rule for identifying a reproducible reference solution; it is not intended to estimate a uniquely optimal or true rank. The criterion selected *Q* = 4 (Results).

Predictive performance was evaluated using repeated bi-cross-validation following Owen and Perry [38]. Rows and columns were each randomly divided into four groups, producing 16 held-out blocks per repetition; four folds were used to retain sufficient taxonomic rows to evaluate ranks up to *Q* = 8. For each block, NMF was fitted only to the retained data, after which the omitted rows and columns were folded onto the fixed factors by non-negative least-squares fitting using 100 multiplicative updates. Held-out root-mean-square error (RMSE) was pooled across the 16 blocks. The procedure was repeated 30 times using identical partitions across candidate ranks, with individual block fits capped at 20,000 iterations. The one-standard-error rule selected the smallest rank whose mean prediction error was no greater than the minimum mean prediction error plus one standard error. Element-wise 10-fold cross-validation with three repetitions was also conducted by assigning a weight of zero to held-out cells; because the held-out cells did not contribute directly to the weighted fitting objective, this analysis was treated only as a supplementary diagnostic and not as a rank-selection criterion (S1 Fig).

Stability of the operational reference solution at *Q* = 4 was further assessed using 500 port-block bootstrap replicates, in which ports were sampled with replacement while all 11 years were retained for each sampled port. Each replicate used 10 random starts. Matched-profile cosine similarity was summarized by its 2.5th, 50th, and 97.5th percentiles and by the proportion of replicate–component matches with cosine similarity ≥ 0.9. These bootstrap distributions were interpreted as perturbation-based stability diagnostics rather than as population confidence intervals. Leave-one-year-out, leave-one-port-out, and leave-one-category-out analyses each used 30 starts; in the leave-one-category-out analysis, the remaining 12 categories were renormalized before fitting. To assess whether the main conclusions depended strongly on the operational reference rank, the same 500-replicate port-block bootstrap and leave-one-year-out, leave-one-port-out, and leave-one-category-out analyses were repeated at *Q* = 5. The *Q* = 5 bootstrap used the same port resamples as the *Q* = 4 bootstrap. As at *Q* = 4, each bootstrap replicate used 10 random starts, whereas each leave-one-year-out, leave-one-port-out, and leave-one-category-out fit used 30 starts. Components were matched to the corresponding full-data representative solution at the same rank using taxonomic-profile cosine similarity.

To assess whether the reference solution depended on the cooperative-years whose audit discrepancies remained unresolved, the *Q* = 4 model was additionally refitted after excluding all 27 cooperative-years classified as open (S2 Table), leaving 457 cooperative-years. These refits used the same specification as the full-data analysis, including the 30 random starts and the consensus diagnostics based on 30 random-initialization fits. Refitted profiles were matched to the full-data representative solution using taxonomic-profile cosine similarity, and normalized component scores were compared over the retained cooperative-years using Spearman correlation.

Sensitivity to data treatment was assessed by fitting raw catch weights and log(1 + y)-transformed catch weights at *Q* = 4 and *Q* = 5. Port-year standardization is inherent in the composition matrix (Eq 2). Standardization by category was not used because it would equalize the weights of categories irrespective of their recorded weights and would amplify categories dominated by recorded zeros; instead, the influence of individual categories was assessed using the leave-one-category-out analysis. At *Q* = 4, recorded-zero cells were additionally assigned weights of 0.5 and 0 while positive cells retained a weight of 1. Component profiles were compared with those of the primary composition model using matched cosine similarity, and the fitted compositions at recorded-zero cells were also summarized. Reconstruction was evaluated globally using squared Euclidean loss and $R_{u}^{\text{2}}$, and locally using the port-year total-variation distance between observed and reconstructed composition and the category-specific mean absolute error. Port-year total-variation distances were summarized overall, by year, and by port, and observed and reconstructed compositions were compared for all 484 port-years (Results).

Spatial smoothing was evaluated only as a sensitivity analysis, using the Gaussian-kernel covariate formulation of Satoh [39,40]. For ports *k* and *l* separated by distance *d*_*kl*_, proximity was defined as $K_{kl}(\beta )=\text{exp}\left(-\beta d_{kl}^{\text{2}}\right)$. The reference bandwidth was based on the median nearest-neighbor distance (σ_0_ = 2.37 km), giving $\beta_{\text{med}}=\text{1}/\left(\text{2}\sigma_{\text{0}}^{\text{2}}\right)=\text{0}.\text{0890 }{km}^{-\text{2}}$ and a half-weight distance of 2.79 km. Sensitivity was examined over seven values, $\beta =\beta_{\text{med}}\cdot {\text{2}}^{r}$, *r* = −3, …, 3. In the non-spatial model, *B = C*, whereas in the spatial model, $B=CA_{\beta }$, where *C* is Q×484 and $A_{\beta }=I_{\text{1}\text{1}}⊗K_{\beta }$; smoothing therefore operated only among ports within the same year. The reference spatial fit used 30 starts, and each of the other six bandwidths used 10 starts. Spatial and non-spatial solutions were compared using matched-profile cosine similarity, Pearson correlations between corresponding unnormalized component scores, and $R_{u}^{\text{2}}$. Because port coordinates represent landing and reporting locations rather than fishing grounds, spatial smoothing was interpreted only as a sensitivity analysis.

Residual spatial structure was assessed separately by year using Moran’s I. The reference kernel at $\beta_{\text{med}}$ was assigned a zero diagonal and row-standardized to form the spatial weight matrix. The 23 tested series comprised the 13 taxonomic shares, the four normalized component scores of each of the non-spatial and spatial models, and the port-year total-variation reconstruction errors of both models. Two-sided P-values were obtained from 9,999 within-year permutations using the permutation distribution of ∣I∣. The resulting 253 tests (23 series × 11 years) were treated as a single multiple-testing family and adjusted using the Benjamini–Hochberg procedure [41].

Finally, normalized component scores were compared with the 13 observed taxonomic shares, the compositional HHI, the maximum taxonomic share, and the log total recorded weight of the 13 categories using Spearman rank correlations across the 484 port-years. For each component, the upper 10% of port-years by normalized score were used to compare mean observed composition, mean reconstructed composition, and the fitted component profile. The highest-scoring port-year for each component and the port-year with the largest total-variation reconstruction error were also examined. Fits with 10 starts (bootstrap replicates and the six non-reference bandwidths) used seeds 1–10; the seeds of fold assignments, bootstrap parts, consensus runs, and permutation tests are listed in S1 Text, and the complete validation outputs are provided in the reproducibility archive.

## 3 Results

### 3.1 Characteristics of the historical catch record

After correction of the verified transcription errors (S1 Table), the 13 analyzed categories accounted for a total recorded catch weight of 58,241,377 kg across the 484 port-years (Table 2). Recorded catch weight was concentrated in a small number of categories. Freshwater clam accounted for 46.0% of the total, followed by Crucian carp (14.2%), Landlocked Ayu (9.8%), Isaza goby (7.7%), Willow gudgeon (7.5%), and Lake prawn (7.0%); the remaining seven categories together accounted for 7.8%. Annual totals were 6.4 million kg in both 1962 and 1963 and ranged from 4.7 to 5.5 million kg in each subsequent year (S3 Table). The five ports with the largest cumulative catches accounted for 52.4% of the total recorded weight over the study period.

**Table 2:** Recorded catch by category over 1962–1972 after correction of the verified transcription errors.

Category	Recorded weight (kg)	Share of total (%)^a^	Port-years with zero (%)^b^	Concentration among reporting ports (range)^c^	Dominant port-years^d^
Trout	281,746	0.5	52.1	0.168 (0.117–0.234)	0
Landlocked Ayu	5,706,766	9.8	24.0	0.103 (0.067–0.136)	78
Common carp	1,468,596	2.5	5.4	0.072 (0.061–0.117)	0
Crucian carp	8,259,800	14.2	0.6	0.071 (0.055–0.131)	170
Japanese dace	448,776	0.8	37.8	0.188 (0.101–0.422)	0
Eel	276,416	0.5	18.2	0.156 (0.045–0.215)	0
Isaza goby	4,467,795	7.7	53.1	0.164 (0.135–0.282)	28
Willow gudgeon	4,377,556	7.5	7.6	0.097 (0.070–0.152)	23
Three-lips	1,301,313	2.2	17.4	0.092 (0.065–0.116)	9
Oriental river prawn	199,920	0.3	68.2	0.202 (0.104–0.353)	0
Lake prawn	4,077,059	7.0	32.4	0.188 (0.162–0.227)	15
Freshwater clam^e^	26,797,213	46.0	47.1	0.147 (0.118–0.181)	161
Pearl mussel	578,421	1.0	76.4	0.284 (0.206–0.861)	0
Total	58,241,377	100.0	—	—	484

^a^Percentage of the total recorded weight across the 13 analyzed categories (58,241,377 kg).

^b^^P^ercentage of the 484 port-years in which the category was recorded as zero; overall, 2,131 of the 6,292 category-by-port-year cells (33.9%) were recorded as zero.

^c^Herfindahl–Hirschman index (HHI) of the category-specific recorded weight across the 44 ports, calculated separately for each year; values are the median and range across the 11 years.

^d^Number of port-years in which the category had the largest share.

^e^Freshwater clam weight is recorded shell-on.

Recorded zeros occurred in 2,131 of the 6,292 cells (33.9%). Category-specific zero rates ranged from 0.6% for Crucian carp to 76.4% for Pearl mussel. Five categories—Freshwater clam, Trout, Isaza goby, Oriental river prawn, and Pearl mussel—were recorded as zero in at least 47% of port-years (Table 2). The zero rate for Freshwater clam was 29.5% in 1962 and at least 52% in every year from 1967 onward.

Port-year composition was typically concentrated in one category. The median compositional HHI was 0.391 (interquartile range [IQR], 0.288–0.587), and the median maximum category share was 0.560 (IQR, 0.425–0.749). A single category accounted for more than 90% of recorded weight in 40 port-years, whereas no category reached 50% in 190 port-years. The dominant category was Crucian carp in 170 port-years, Freshwater clam in 161, Landlocked Ayu in 78, Isaza goby in 28, Willow gudgeon in 23, Lake prawn in 15, and Three-lips in 9. Category-specific concentration across reporting ports ranged from relatively high for Pearl mussel (median HHI, 0.284) to relatively low for Crucian carp (0.071; Table 2). Composition also varied between consecutive years within ports: the median total-variation distance between successive observations for the same port was 0.150 (IQR, 0.076–0.240; 440 port-year pairs).

### 3.2 Rank assessment

Reconstruction improved monotonically with rank, with *R*_u_^2^ increasing from 0.452 at *Q* = 1 to 0.994 at *Q* = 8 (Table 3; Fig 2). Bi-cross-validation error also decreased monotonically, reaching its minimum at *Q* = 8 (0.1561 ± 0.0007); the one-standard-error rule selected *Q* = 7 (threshold, 0.15677). Element-wise cross-validation did not provide a usable rank-selection criterion because fitted values at zero-weight held-out cells were unstable, resulting in RMSE values far outside the 0–1 range of the compositional data at *Q* ≥ 2 (S1 Fig). All 30 starts converged at every rank. Solutions were reproducible across starts through *Q* = 5, whereas component correspondence deteriorated sharply at *Q* ≥ 6, with minimum matched cosine ≤ 0.026 and PAC 0.071–0.098 for *Q* = 6–8. The effective rank of the composition matrix was 7.77.

**Table 3:** Rank diagnostics for *Q* = 1–8.

*Q*	R_u_^2^^a^	BCV mean ± SE^b^	Cophenetic^c^	PAC^c^	min cosine^d^	Objective range^d^	Converged^e^
1	0.452	0.1817 ± .0002	—	—	1	0	30/30
2	0.761	0.1764 ± .0005	1	0	1	0	30/30
3	0.898	0.1697 ± .0005	1	0	1	0	30/30
4	0.937	0.1646 ± .0008	1	0	1	0.002	30/30
5	0.961	0.1612 ± .0008	0.9988	0.0117	0.9901	0.484	30/30
6	0.977	0.1584 ± .0007	0.9954	0.0963	0.0005	1.964	30/30
7	0.988	0.1566 ± .0008	0.9935	0.098	0.0002	1.997	30/30
8	0.994	0.1561 ± .0007	0.9934	0.0712	0.0256	2.050	30/30

^a^*R*_u_^2^, uncentered *R*^2^ of the representative fit with the smallest final objective value among 30 random starts.

^b^Held-out RMSE from repeated bi-cross-validation in which rows and columns were each partitioned into four folds (16 held-out blocks per repetition; mean ± standard error over 30 repetitions).

^c^Cophenetic correlation coefficient calculated from consensus matrices based on 30 random-initialization fits; undefined at *Q* = 1. PAC was calculated from the same consensus matrices using an ambiguity interval of 0.1–0.9.

^d^Agreement among the 30 full-data random starts, summarized by the minimum matched-profile cosine similarity after component alignment and by the range of final objective values across starts.

^e^Number of starts meeting the convergence criterion within 100,000 iterations.

**Fig 2:**
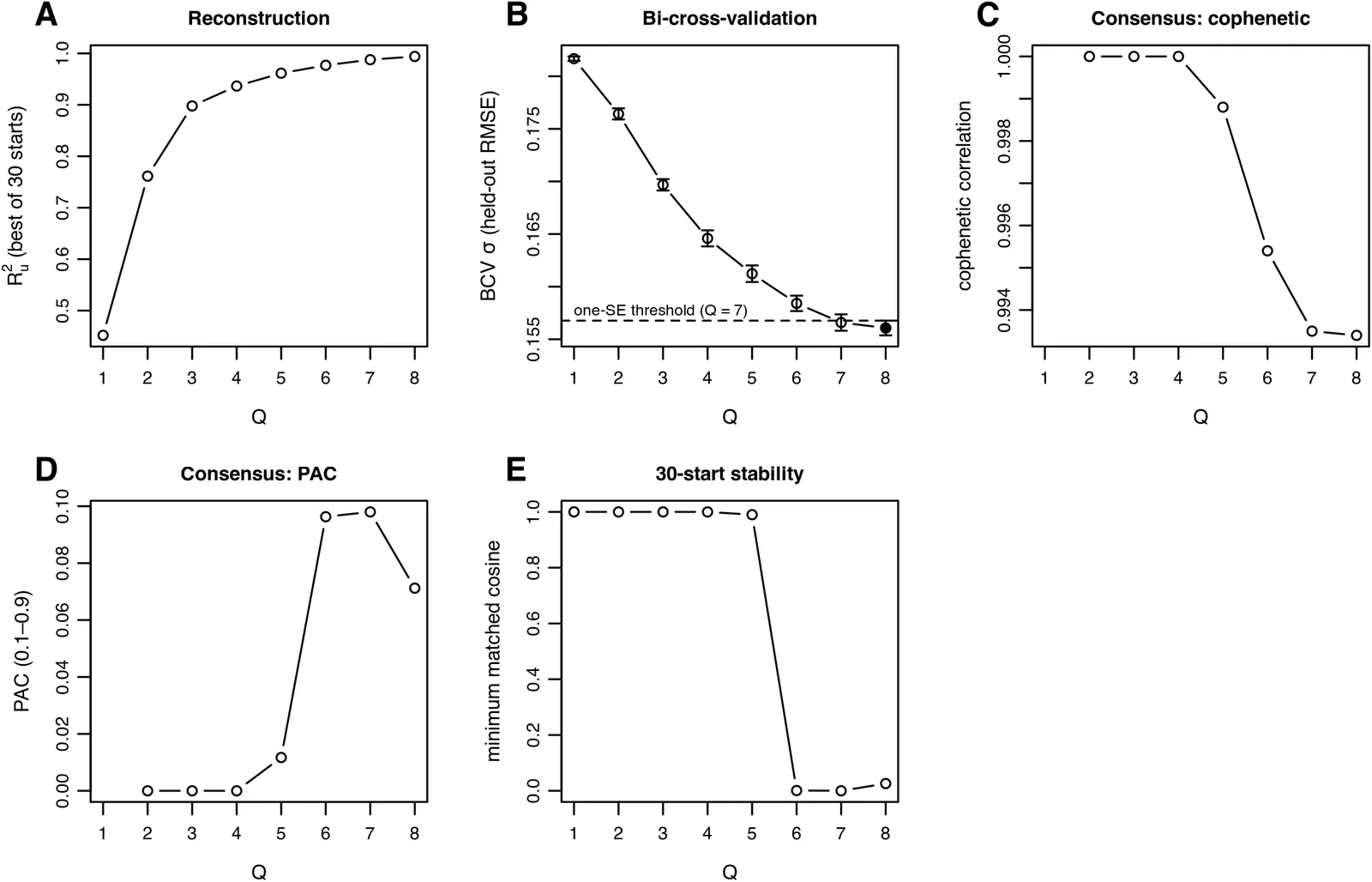
Rank diagnostics for *Q* = 1–8. **A** Uncentered *R*_u_^2^ of the representative fit with the smallest final objective value among 30 random starts. **B** Held-out RMSE from repeated four-fold bi-cross-validation (mean ± standard error over 30 repetitions); the dashed line indicates the one-standard-error threshold and the filled symbol indicates the minimum. **C** Cophenetic correlation coefficient. **D** Proportion of ambiguous clustering (PAC; ambiguity interval, 0.1–0.9) from consensus matrices based on 30 random-initialization fits; undefined at *Q* = 1. **E** Minimum matched-profile cosine similarity among the 30 full-data starts. Values are reported in Table 3.

The reference-rank criterion defined in the Methods—all starts converged, minimum matched cosine equal to 1.000 after rounding to three decimal places, and PAC ≤ 0.005—identified *Q* = 4 as the largest qualifying rank. The four *Q* = 4 components reappeared at *Q* = 5 with matched cosine similarities of 0.990, 1.000, 0.910, and 1.000. The additional *Q* = 5 component was dominated by Willow gudgeon (0.750), with a secondary contribution from Three-lips (0.214). Predictive criteria thus favored higher ranks, whereas cross-start component correspondence remained high through *Q* = 5 but deteriorated sharply at *Q* ≥ 6. Because these criteria did not support a common range of ranks, the exact rank was treated as unresolved, and *Q* = 4 was used as the operational reference rank rather than as a selected optimum.

### 3.3 Reference solution (*Q* = 4): Component profiles, scores, and reconstruction

Because the exact rank remained unresolved, the *Q* = 4 solution was used as the operational reference defined in the Methods rather than as a uniquely selected model. The representative *Q* = 4 solution had a squared Euclidean loss of 13.86466 and an $R_{u}^{\text{2}}$ of 0.9365. Three of the four component profiles were dominated by a single category (Fig 3). C1 was dominated by Crucian carp (0.711), with smaller contributions from Common carp (0.105) and Willow gudgeon (0.095). C2 was dominated by Freshwater clam (0.947), and C4 by Landlocked Ayu (0.825). C3 was a mixed profile, with no category exceeding 0.38: Isaza goby contributed 0.375, Lake prawn 0.305, and Willow gudgeon 0.262. Because Freshwater clam is recorded shell-on, its contribution to C2 refers to shell-on recorded weight. Normalized component scores across port-years are shown in Fig 4 and mapped to port locations in Fig 5. These scores are continuous weights rather than class memberships.

**Fig 3:**
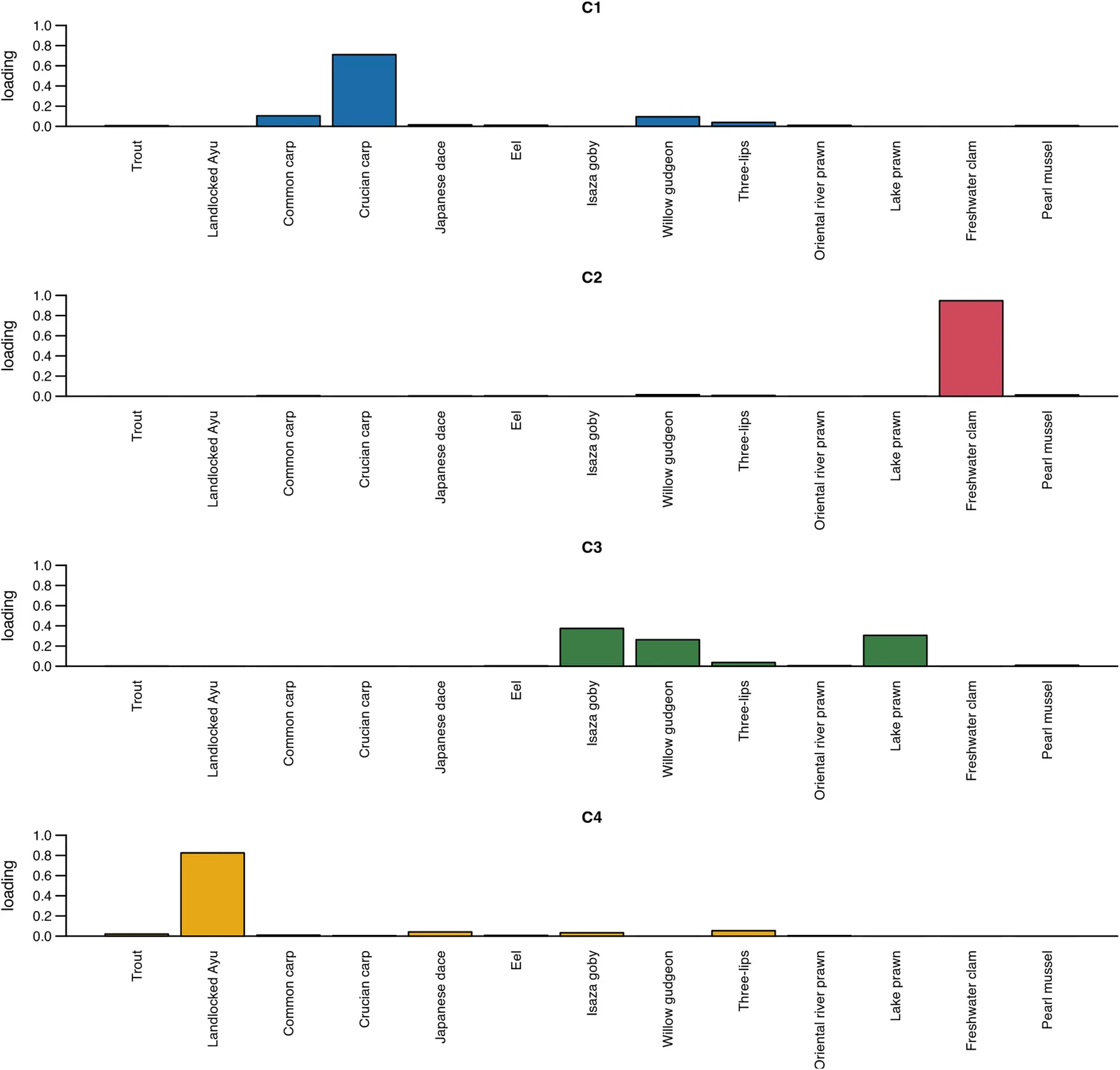
Taxonomic profiles of the four components of the *Q* = 4 reference solution. Bars show the loading of each of the 13 categories in components C1–C4 (columns of X); loadings within each component sum to one. Category labels are those of Table 1.

**Fig 4:**
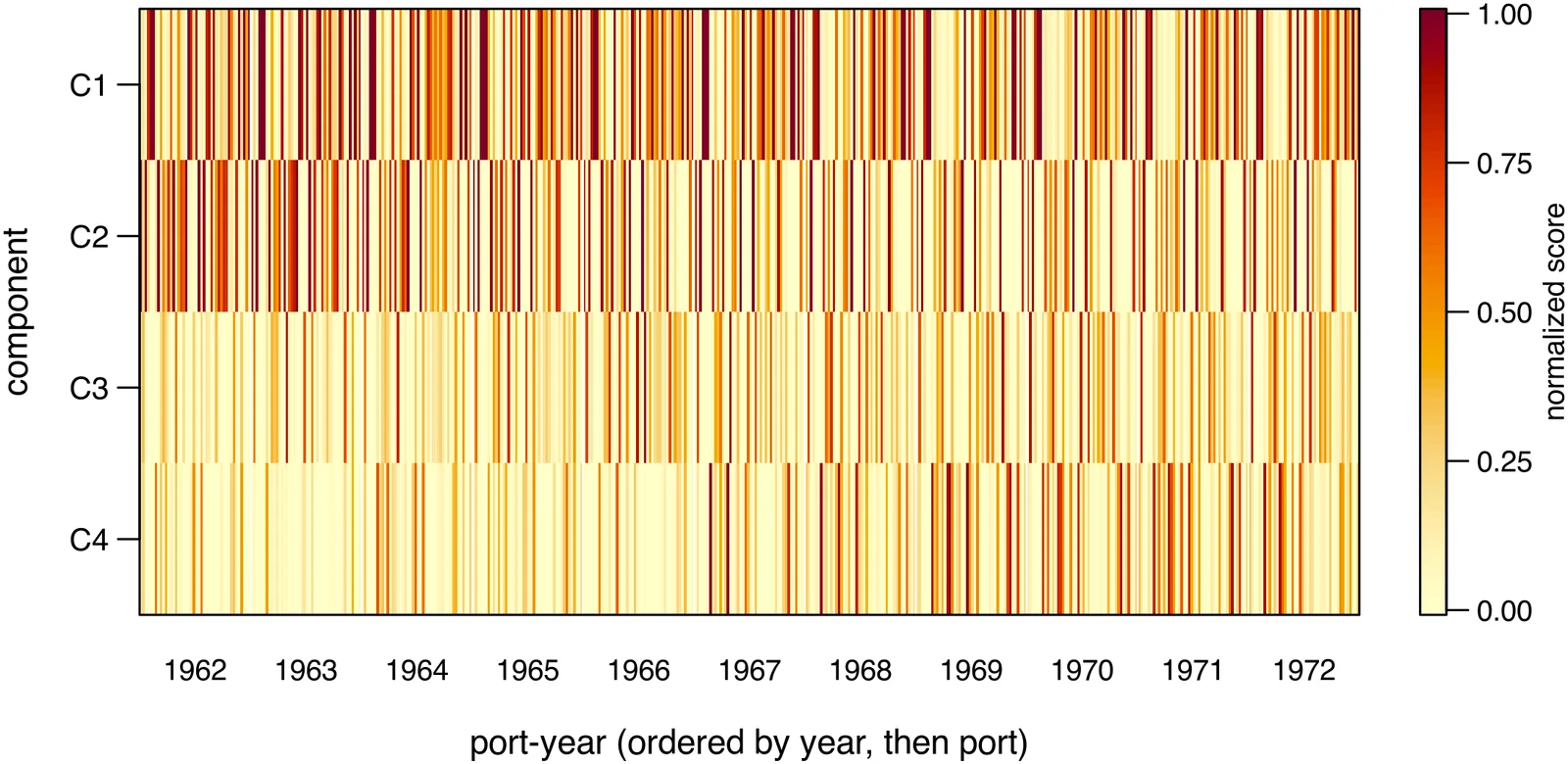
Normalized component scores of all 484 port-years. Heat map of the scores of C1–C4 (rows) for each port-year (columns, ordered by year and, within each year, alphabetically by port; white lines separate years). Scores were normalized to sum to one within each port-year; the color key gives the normalized score.

**Fig 5:**
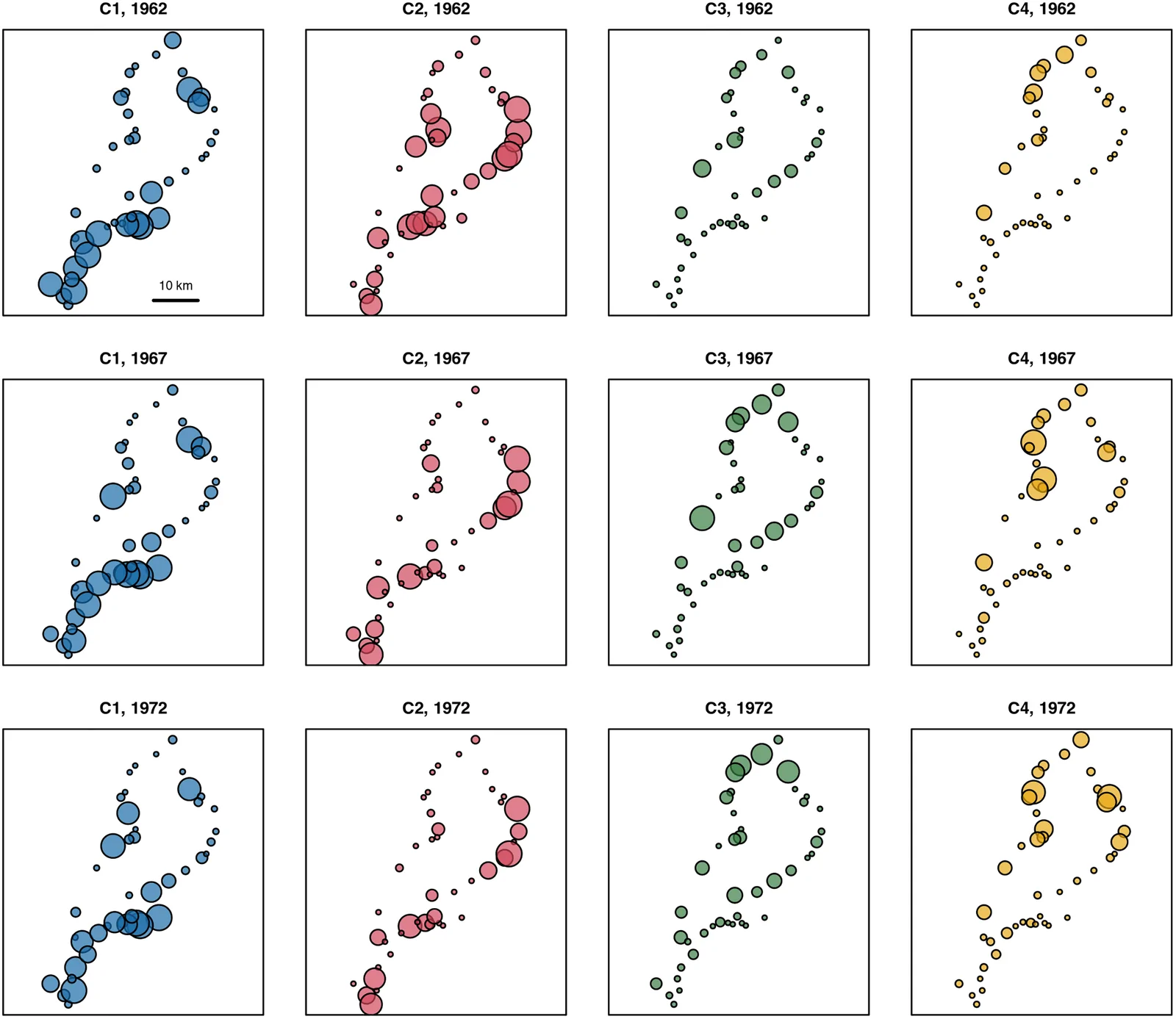
Normalized component scores at port locations in 1962, 1967, and 1972. Each panel shows the 44 ports in projected coordinates (JGD2011 / Japan Plane Rectangular CS VI). Symbol size increases with the normalized score for the component (C1–C4, columns) in the indicated year (rows). Ports represent landing and reporting locations, not fishing grounds. Scale bar, 10 km.

Local reconstruction accuracy varied across port-years (Fig 6). The median total-variation distance between observed and reconstructed port-year compositions was 0.140 (IQR, 0.082–0.210), with a maximum of 0.620 for Yoshikawa in 1972, which was dominated by Three-lips. Total-variation distance exceeded 0.30 in 43 port-years. The annual median was 0.102 in 1962 and 0.175 in 1972, with values ranging from 0.106 to 0.161 in the intervening years. Port-level medians ranged from 0.036 for Ayame to 0.457 for Yoshikawa and exceeded 0.20 for 11 of the 44 ports. Of the 43 port-years with total-variation distances above 0.30, 27 belonged to five ports: Yoshikawa, Shima, Sabae, Komatsu, and Notogawa. Category-specific mean absolute error ranged from 0.007 for Eel to 0.060 for Willow gudgeon. Observed and reconstructed compositions for the highest-scoring port-year of each component and for Yoshikawa in 1972 are shown in Fig 7.

**Fig 6:**
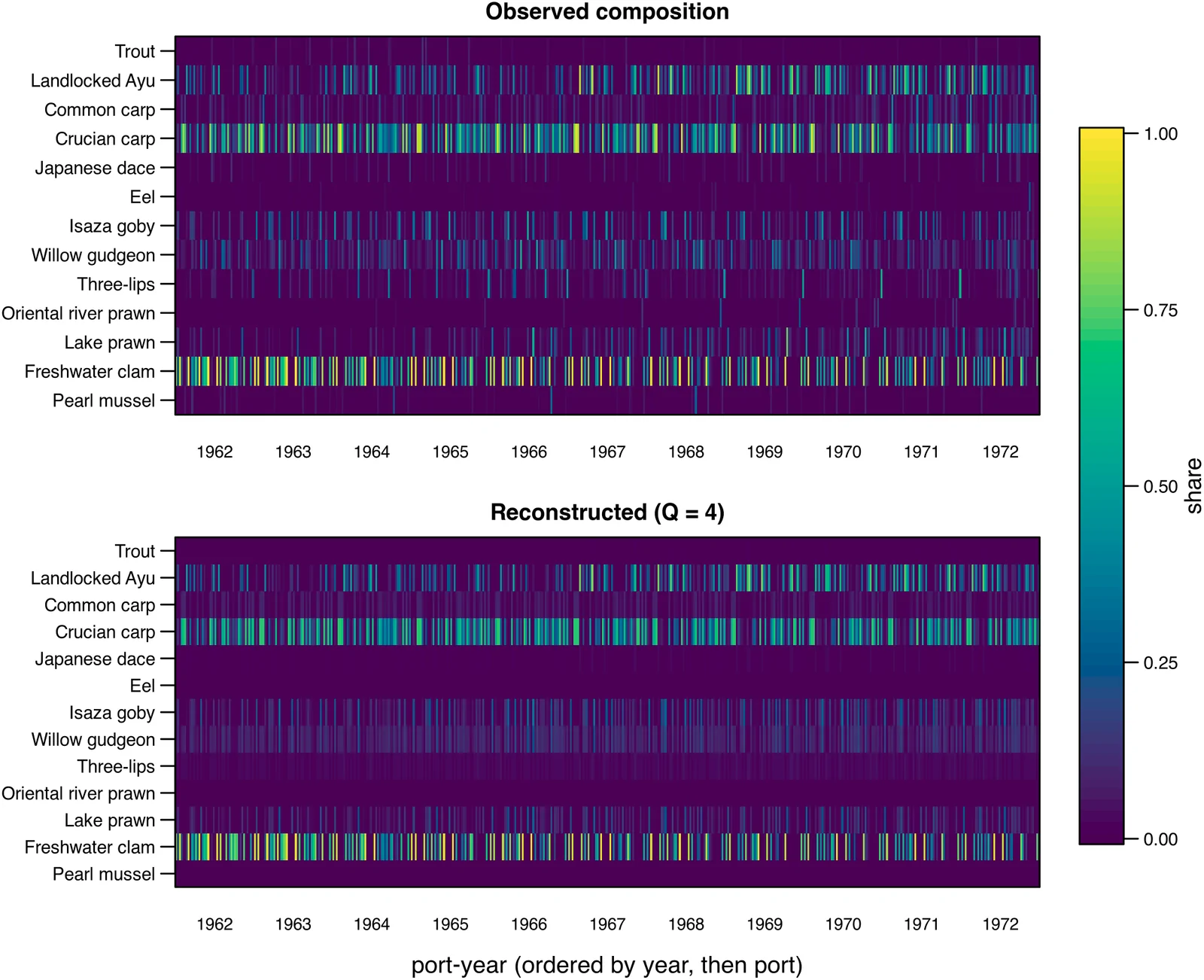
Observed and reconstructed catch composition of all 484 port-years. Upper panel, observed composition (P); lower panel, reconstruction by the *Q* = 4 reference solution (XB, renormalized to sum to one within each port-year). Rows are the 13 categories and columns are port-years ordered by year and, within each year, alphabetically by port; the color key gives the share.

**Fig 7:**
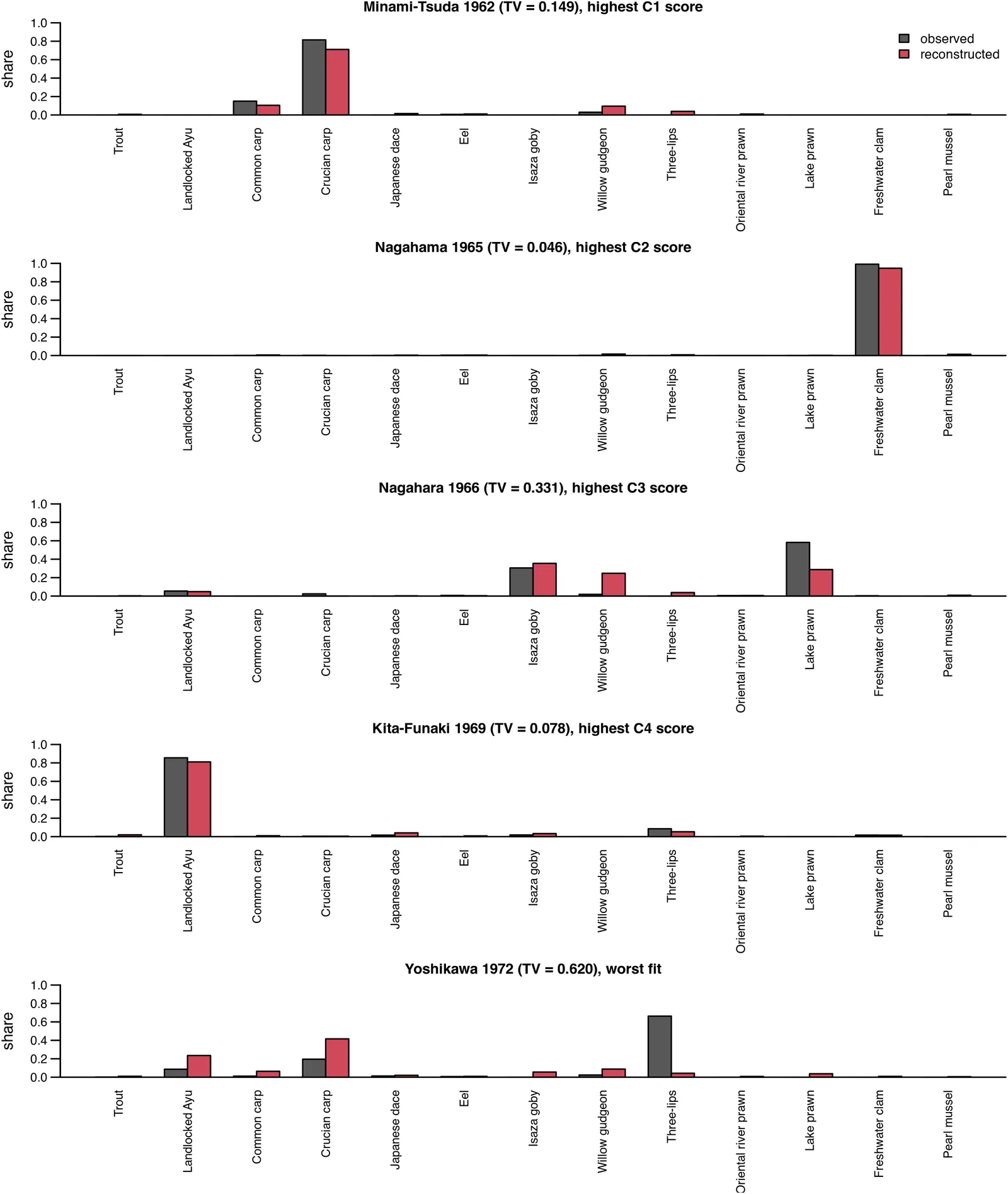
Observed and reconstructed composition of exemplar port-years. The figure shows the highest-scoring port-year for each component (Minami-Tsuda 1962 for C1, Nagahama 1965 for C2, Nagahara 1966 for C3, and Kita-Funaki 1969 for C4) and the port-year with the largest reconstruction error (Yoshikawa 1972). Bars show observed (gray) and reconstructed (red) shares of the 13 categories. TV denotes the total-variation distance between observed and reconstructed composition.

Three components corresponded strongly to a single observed taxonomic share (Table 4). Spearman correlations between normalized component scores and their defining shares were 0.985 for C1 and Crucian carp, 0.941 for C2 and Freshwater clam, and 0.967 for C4 and Landlocked Ayu. Among the upper 10% of port-years by component score, the mean observed share of the defining category was 0.794, 0.924, and 0.617 for C1, C2, and C4, respectively. C3 did not show comparably strong correspondence with any single taxonomic share. Its highest correlations were with Lake prawn (0.703), Isaza goby (0.687), and Willow gudgeon (0.613). Higher C3 scores were also associated with lower compositional HHI (−0.704) and lower maximum category share (−0.704), consistent with more mixed observed compositions.

**Table 4:** Spearman rank correlations between normalized component scores of the *Q* = 4 solution and direct summaries across the 484 port-years.

Direct summary^a^	C1^b^	C2^b^	C3^b^	C4^b^
Trout	−0.116	−0.201	0.193	0.496
Landlocked Ayu	−0.434	−0.238	0.360	0.967
Common carp	0.751	−0.287	−0.225	−0.242
Crucian carp	0.985	−0.463	−0.273	−0.351
Japanese dace	−0.037	−0.071	0.010	0.373
Eel	0.182	0.002	−0.077	0.018
Isaza goby	−0.513	−0.149	0.687	0.521
Willow gudgeon	0.225	−0.168	0.613	−0.064
Three-lips	−0.059	0.015	0.225	0.426
Oriental river prawn	−0.084	0.062	0.252	0.100
Lake prawn	−0.379	−0.098	0.703	0.425
Freshwater clam	−0.495	0.941	−0.252	−0.286
Pearl mussel	−0.022	0.415	−0.066	−0.400
Compositional HHI	−0.029	0.181	−0.704	−0.490
Maximum share	−0.036	0.201	−0.704	−0.458
log total recorded weight (13 categories)	−0.566	0.449	0.193	0.033

The direct summaries comprise the observed shares of the 13 taxonomic categories, compositional HHI, maximum taxonomic share, and log total recorded weight across the 13 categories in each port-year. Component scores were normalized to sum to one within each port-year, and each correlation was calculated across the 484 port-years.

^a^Observed shares of the 13 taxonomic categories, the compositional Herfindahl–Hirschman index (HHI), the maximum taxonomic share, and the log total recorded weight of the 13 categories in each port-year.

^b^Component scores normalized to sum to one within each port-year; each correlation was calculated across the 484 port-years.

### 3.4 Stability and sensitivity of the reference solution

In the port-block bootstrap analysis (500 replicates), the 2.5th percentiles of matched-profile cosine similarity were 0.993, 0.999, 0.135, and 0.906 for C1–C4, respectively; the corresponding medians were 0.999, 1.000, 0.958, and 0.997. Overall, 92.9% of replicate-component matches had cosine similarity ≥ 0.9. C1, C2, and C4 therefore showed consistently high correspondence across port resamples, whereas C3 showed substantially lower correspondence in the lower tail of the bootstrap distribution. Leave-one-year-out and leave-one-port-out analyses reproduced all four components, with minimum cosine similarities of 0.999 and 0.978, respectively; both minima occurred for C3.

In the leave-one-category-out analyses, removal of the defining category produced the largest changes in the corresponding taxon-dominated component. C3 also changed when Crucian carp was removed. Removal of any of the other ten categories left all components with cosine similarities ≥ 0.989 (Table 5).

**Table 5:** Stability, sensitivity, and spatial diagnostics of the *Q* = 4 reference solution.

Analysis	C1	C2	C3	C4	Other
Bootstrap (500 port-block replicates): 2.5th / 50th / 97.5th percentile of matched cosine	0.993 / 0.999 / 1.000	0.999 / 1.000 / 1.000	0.135 / 0.958 / 0.998	0.906 / 0.997 / 1.000	92.9% of replicate–component matches ≥ 0.9
Leave-one-year-out: minimum cosine	0.9999	1.000	0.9988	0.9999	11 fits
Leave-one-port-out: minimum cosine	0.9998	1.000	0.9777	0.999	44 fits; C3 minimum when Asahi excluded
Leave-one-category-out: minimum cosine	0.7359 (Crucian carp removed)	0.7583 (Freshwater clam removed)	0.5597 (Crucian carp removed)	0.2037 (Landlocked Ayu removed)	13 fits; removal of any of the other ten categories ≥ 0.989
Raw weights, *Q* = 4: cosine vs composition	0.992	1.000	0.936	0.987	*Q* = 5: 0.964 / 1.000 / 0.999 / 0.877 / 0.261
log(1 + y) weights, *Q* = 4: cosine vs composition	0.751	0.950	0.828	0.346	*Q* = 5: 0.341 / 0.948 / 0.545 / 0.864 / 0.566
Zero-cell weight 0.5: cosine vs weight 1	1.000	1.000	0.998	1.000	fitted share at zero cells: median 0.009, 90th percentile 0.036, maximum 0.314
Zero-cell weight 0: cosine vs weight 1	0.996	1.000	0.981	0.995	median 0.010, 90th percentile 0.249, maximum 0.988
Spatial model at $\beta_{\text{med}}$: profile cosine / score correlation^a^	0.9995 / 0.916	0.9994 / 0.850	0.9983 / 0.946	0.9996 / 0.937	spatial $R_{u}^{\text{2}}=\text{0}.\text{8}\text{2}\text{2}$ vs non-spatial $R_{u}^{\text{2}}=\text{0}.\text{9}\text{3}\text{7}$; across $\beta_{\text{med}}{\text{2}}^{r}$(r=−3,…,3): minimum matched-profile cosine 0.967–0.99995, $R_{u}^{\text{2}}$ 0.654–0.933
Moran’s I of normalized scores, non-spatial model^b^	0.239 (3/11)	0.101 (0/11)	0.269 (6/11)	0.387 (8/11)	selected observed shares: Isaza goby 0.541 (9/11), Landlocked Ayu 0.391 (7/11), Freshwater clam 0.105 (0/11)
Moran’s I of port-year TV reconstruction error^b^	—	—	—	—	non-spatial −0.069 (0/11); spatial 0.120 (0/11); 95 of 253 tests significant overall

Values for C1–C4 are matched-profile cosine similarities between component profiles unless stated otherwise.

^a^Profile cosine similarity relative to the non-spatial model / Pearson correlation between corresponding unnormalized component scores.

^b^Mean Moran’s I over the 11 years; values in parentheses indicate the number of years significant after Benjamini–Hochberg adjustment across all 253 tests.

Excluding all 27 cooperative-years that remained open after source verification (S2 Table) likewise left the reference solution essentially unchanged. Refitted on the 457 retained cooperative-years, the four component profiles matched the full-data solution with cosine similarities of 0.99999, 0.99999, 0.99779, and 0.99999 for C1–C4, respectively (S5 Table). The dominant category of every component was unchanged, and Spearman correlations of the normalized component scores over the retained cooperative-years were 0.9997, 0.9977, 0.9991, and 0.9986 for C1–C4, respectively. The refit also satisfied the study-specific stability criterion (all 30 starts converged; minimum cross-start matched cosine, 1.000 after rounding to three decimal places; PAC, 0.000), with an uncentered $R_{u}^{\text{2}}$ of 0.9366 (S5 Table).

Fitting raw catch weights rather than compositional shares yielded matched cosine similarities of 0.992, 1.000, 0.936, and 0.987 relative to the composition-based solution at *Q* = 4. At *Q* = 5, four correspondences ranged from 0.877 to 1.000, whereas the fifth was 0.261. For log(1 + y)-transformed weights, the corresponding values were 0.751, 0.950, 0.828, and 0.346 at *Q* = 4, and 0.341, 0.948, 0.545, 0.864, and 0.566 at *Q* = 5. C2 therefore retained the highest correspondence with the composition-based solution under both alternative scalings, whereas correspondence for the other components was lower under the log transformation.

Down-weighting recorded zeros had little effect on the composition-based component profiles. Component cosine similarities were 1.000, 1.000, 0.998, and 1.000 when the zero-cell weight was 0.5 and 0.996, 1.000, 0.981, and 0.995 when it was 0. However, fitted normalized shares at recorded-zero cells were substantially more dispersed when the zero-cell weight was 0 (90th percentile, 0.249; maximum, 0.988) than when it was 0.5 (90th percentile, 0.036; maximum, 0.314) (Table 5).

Repeating the resampling and exclusion analyses at *Q* = 5 produced a similar qualitative pattern. Three of the five *Q* = 5 components were strongly reproducible in the port-block bootstrap, with 2.5th-percentile matched cosine similarities of 0.987, 0.999, and 0.988, whereas the remaining two components had lower values of 0.580 and 0.296. Overall, 92.5% of replicate-component matches had cosine similarity ≥ 0.9. The minimum matched cosine was 0.9987 in the leave-one-year-out analysis and 0.9898 in the leave-one-port-out analysis. Leave-one-category-out perturbations were again more influential, with a minimum matched cosine of 0.210. Thus, increasing the fitted rank from *Q* = 4 to *Q* = 5 preserved a reproducible core structure, but the five-component solution was not uniformly stable under resampling (S4 Table).

### 3.5 Spatial sensitivity and residual spatial structure

Across the seven bandwidths, the minimum matched cosine similarity between spatial and non-spatial component profiles ranged from 0.967 ($\beta =\text{0}.\text{0}\text{1}\text{1}\text{1}\;{\text{km}}^{-\text{2}}$; half-weight distance, 7.9 km) to 0.99995 ($\beta =\text{0}.\text{7}\text{1}\text{1}\text{8}\;{\text{km}}^{-\text{2}}$; half-weight distance, 0.99 km). The $R_{u}^{\text{2}}$ values of the spatial fits ranged from 0.654 to 0.933, compared with 0.937 for the non-spatial model (Table 5). At the reference bandwidth ($\beta =\text{0}.\text{0}\text{8}\text{9}\text{0}\;{\text{km}}^{-\text{2}}$; half-weight distance, 2.79 km), the component profiles closely matched those of the non-spatial model, with cosine similarities of 0.9995, 0.9994, 0.9983, and 0.9996. Correlations between corresponding unnormalized component scores were 0.916, 0.850, 0.946, and 0.937, and $R_{u}^{\text{2}}$was 0.822. Spatial smoothing based on reporting locations therefore did not materially alter the taxonomic profiles but reduced overall reconstruction fit.

Spatial autocorrelation among the observed taxonomic shares was strongest for Isaza goby (mean Moran’s I across years, 0.541; significant after Benjamini–Hochberg adjustment in 9 of 11 years) and Landlocked Ayu (0.391; 7 of 11 years). Crucian carp showed weaker but detectable autocorrelation (0.215; 3 of 11 years), whereas Freshwater clam showed no significant spatial autocorrelation after adjustment (0.105; 0 of 11 years). Normalized scores from the non-spatial model showed a similar pattern: mean Moran’s I was 0.387 for C4 (significant in 8 of 11 years), 0.269 for C3 (6 of 11 years), 0.239 for C1 (3 of 11 years), and 0.101 for C2 (0 of 11 years). These results indicate that spatial autocorrelation was present in some observed taxonomic shares and their corresponding component scores, although its strength varied among components.

The port-year total-variation reconstruction error showed no significant spatial autocorrelation in any year for either model. Mean Moran’s I was −0.069 for the non-spatial model and 0.120 for the spatial model, with 0 of 11 years significant in both cases. Overall, 95 of the 253 tests remained significant after adjustment, all involving observed taxonomic shares or component scores. Thus, although observed taxonomic shares and component scores exhibited spatial autocorrelation, the magnitude of port-year reconstruction error showed no detectable spatial autocorrelation at the scale of the reference kernel.

## 4 Discussion

### 4.1 Does the record support a stable latent structure? Reproducible components and their limits

The cooperative-level record supports a reproducible core of catch-composition patterns, but not a unique and uniformly stable latent structure. Three components of the *Q* = 4 reference solution—dominated by Crucian carp, Freshwater clam, and Landlocked Ayu—were reproduced across random starts, port-block resampling, and leave-one-year-out and leave-one-port-out analyses. The mixed component comprising Isaza goby, Lake prawn, and Willow gudgeon was less stable, with substantially lower correspondence in the lower tail of the bootstrap distribution. Importantly, repeating the resampling and exclusion analyses at *Q* = 5 yielded the same qualitative pattern: three components remained strongly reproducible, whereas two were less stable. Thus, the main conclusion that the record contains a reproducible core of catch-composition patterns does not depend strongly on using *Q* = 4 rather than *Q* = 5, although the less stable components are rank-dependent.

The record did not, however, resolve a unique number of latent patterns. Bi-cross-validation reached its minimum at *Q* = 8 and the one-standard-error rule selected *Q* = 7, whereas component correspondence across random starts deteriorated sharply at *Q* ≥ 6. Accordingly, *Q* = 4 was used only as an operational reference under the study-specific stability rule defined in the Methods, not as an estimate of a true or uniquely optimal rank. The *Q* = 5 solution retained the main *Q* = 4 profiles while adding a component dominated by Willow gudgeon, with a secondary contribution from Three-lips, but not all *Q* = 5 components were uniformly stable under resampling. Increasing the fitted rank therefore added descriptive flexibility without resolving the underlying rank uncertainty.

The reproducible components also depended on how recorded catch magnitude was represented. Under raw catch weights, the three category-dominant components remained closely matched to the composition-based solution, whereas the mixed component showed somewhat lower correspondence. Under the log transformation, correspondence declined more substantially for several components, particularly the Ayu-dominated component. The reproducible structure identified here should therefore be interpreted specifically as a representation of recorded catch composition under the primary weighting and normalization scheme rather than as a structure invariant to data transformation.

These findings complement previous analyses of aggregated Lake Biwa catch series [17,18] by showing both the possibilities and limits of extracting multivariate patterns from cooperative-level records. The recurrence of several component profiles across perturbations shows that some patterns in the historical record are statistically reproducible. However, reproducibility of the statistical representation does not establish an underlying biological mechanism. Because the data contain no direct information on fishing effort, gear, or fishing grounds, the observed patterns cannot be attributed separately to biological change, fishing activity, or reporting processes (Section 4.4).

### 4.2 What NMF added beyond direct catch summaries

The direct summaries already captured much of the dominant structure in the record. A small number of categories accounted for most recorded weight, and many port-years were dominated by a single category. Consistent with these summaries, three NMF components corresponded closely to the observed shares of Crucian carp, Freshwater clam, and Landlocked Ayu (Spearman correlations, 0.985, 0.941, and 0.967, respectively). For these category-dominant components, NMF therefore added relatively little beyond the corresponding taxonomic shares. C3 differed in summarizing a mixture of Lake prawn, Isaza goby, and Willow gudgeon associated with lower compositional concentration, but it was also the least stable component. Thus, the aspect of the NMF representation that extended most clearly beyond the marginal shares received weaker support from the data.

NMF nevertheless provided a common low-dimensional representation of all 484 port-years that could be evaluated explicitly for stability and reconstruction accuracy. Within this framework, rank uncertainty, reproducibility under resampling and refitting, sensitivity to analytical choices, and local reconstruction error could be assessed together. Overall reconstruction fit was high ($R_{u}^{\text{2}}\approx \text{0}.\text{9}\text{3}\text{7}$), but the median port-year total-variation error was 0.140, and 43 port-years had errors above 0.30. The low-dimensional representation therefore summarized the record well overall without reproducing every port-year equally well.

In this dataset, NMF was therefore most informative as a tool for compact summarization and explicit stability assessment rather than as evidence for substantial latent structure beyond that already visible in the direct summaries. Its principal contribution was to distinguish reproducible patterns from unstable ones and to identify where a compact representation no longer approximated the observed composition adequately. The mixed component and the exact rank—the features that extended furthest beyond the direct summaries—were also those for which the record provided the weakest support.

### 4.3 Spatial structure at reporting locations

Spatial structure was evident in some observed catch shares and was reflected in the non-spatial NMF representation. Isaza goby and Landlocked Ayu showed significant spatial autocorrelation among reporting ports in most years, whereas Freshwater clam showed none. Normalized scores from the non-spatial solution showed a corresponding pattern, with the strongest autocorrelation for the Landlocked Ayu component and no significant autocorrelation for the Freshwater clam component. Adding a Gaussian proximity kernel changed the component profiles very little across the seven bandwidths spanning a 64-fold range, but reduced reconstruction fit at the reference bandwidth ($R_{u}^{\text{2}}=\text{0}.\text{8}\text{2}\text{2}$ versus 0.937 for the non-spatial model). Moreover, port-year reconstruction error showed no significant spatial autocorrelation in any year. Taken together, these results indicate that spatial structure already present in the observed compositions was reflected in the non-spatial components, whereas explicit spatial smoothing did not improve reconstruction fit.

This spatial structure should be interpreted as similarity among reporting locations rather than as evidence of species distributions or habitat structure. The coordinates identify the ports at which cooperatives landed and reported catches, not the fishing grounds from which those catches originated. Neighboring ports could therefore have reported catches from overlapping or distant fishing grounds, and similarity in recorded composition could reflect shared fishing grounds, gear use, market conditions, or reporting practices. Because these mechanisms cannot be separated using the historical record, the spatial analyses are interpreted as descriptions of reporting-location structure and as sensitivity checks on the NMF representation.

### 4.4 Limitations of the record and scope of inference

The principal limitation is that recorded catch composition cannot be interpreted as biological abundance or species distribution. The record contains no information on fishing effort, gear, or fishing grounds, so differences among ports or years may reflect changes in biological abundance, fishing activity, market conditions, or reporting practices. Likewise, the port associated with each cooperative is a landing and reporting location rather than a fishing ground, and neighboring ports may represent catches taken from overlapping or distant areas. The components identified here therefore describe patterns of recorded catch composition at reporting locations, not the composition or spatial distribution of fish stocks.

The historical categories, measurements, and source records introduce additional uncertainty. Trout and Willow gudgeon are composite historical categories that do not map one-to-one onto currently recognized species; 33.9% of analytical cells are recorded as zero, but the underlying meaning of these zeros cannot be recovered; and Freshwater clam, which accounts for 46.0% of recorded weight, is reported as shell-on weight rather than soft-tissue biomass or abundance. The source also contains internal inconsistencies: of the 45 cooperative-years flagged by the audit, 18 were resolved through primary-source verification and 27 remain open. Sensitivity and exclusion analyses indicate that the main reference profiles are not driven by individual years, ports, alternative treatments of recorded zeros, or the 27 cooperative-years that remained open after source verification (S5 Table), although these diagnostics cannot verify the accuracy of the retained historical values themselves.

The temporal and statistical scope of inference is also limited to the observed 1962–1972 record. The dataset contains 44 reporting units in each of 11 years, and the bootstrap distributions quantify stability under resampling of these historical observations rather than population uncertainty. Similarly, the permutation tests characterize spatial structure within the observed configuration of reporting ports. The analysis therefore does not estimate changes after 1972 or identify causal effects of the Lake Biwa Comprehensive Development Project.

Within these limits, the study provides a reproducible reference description of cooperative-level recorded catch composition for 1962–1972. The category-dominant components, the less stable mixed component, the unresolved rank, and the reconstruction diagnostics together identify which features of the historical record are reproducible and which remain uncertain. Comparable later data cross-classified by cooperative and catch category—and ideally accompanied by information on effort, gear, fishing grounds, and markets—would allow these patterns to be evaluated over time using the same procedures. Such comparisons are currently constrained because later official statistics do not provide a directly comparable cooperative-by-category series, consistent with the broader scarcity and aggregation of historical inland-fisheries records [5–12].

## 5 Conclusion

The 1962–1972 cooperative-level catch record of Lake Biwa supports a reproducible core of recorded catch-composition patterns, but not a unique and uniformly stable latent structure. Three category-dominant components—Crucian carp, Freshwater clam, and Landlocked Ayu—were reproducible across random starts, port resampling, and exclusion of individual years or ports, and the same qualitative conclusion persisted at *Q* = 5. In contrast, mixed or additional components were less stable, and predictive and stability criteria did not support a common rank. The principal contribution of NMF was therefore not to reveal wholly new patterns beyond the marginal taxonomic shares, but to provide a common framework for evaluating component reproducibility, rank uncertainty, sensitivity to data representation, and reconstruction error. Spatial analyses further showed that some observed shares and component scores were autocorrelated among reporting ports, whereas reconstruction error showed no detectable spatial autocorrelation at the scale examined.

These results should be interpreted as a reproducible description of the historical catch record rather than as direct evidence of biological or fishing conditions. Recorded catch composition cannot be equated with stock abundance or species distribution because the data contain no information on fishing effort, gear, or fishing grounds, and the spatial coordinates identify landing and reporting locations. Within these limits, the dataset provides a documented historical reference for cooperative-level recorded catch composition during 1962–1972 and establishes which features of its low-dimensional representation are reproducible and which remain uncertain. Comparable later records with similar taxonomic, reporting-location, and temporal resolution would allow these patterns to be evaluated over time.

## Supporting information

S1 Text Supplementary methods for data verification, validation, sensitivity analyses, and reproducibility. Provides additional details on the transcription and verification of the historical catch records; construction of the 13-category analytical matrix; treatment of recorded zeros; NMF fitting, component matching, and rank diagnostics; bi-cross-validation and element-wise cross-validation; port-block bootstrap and leave-one-year-, port-, and category-out analyses at *Q* = 4 and *Q* = 5; sensitivity to raw and log(1 + y)-transformed catch weights and zero-cell weighting; spatial smoothing and Moran’s I analyses; random-seed conventions; and the organization of the reproducibility archive.
(DOCX)

S1 Fig Element-wise cross-validation as a supplementary diagnostic. Held-out RMSE from element-wise 10-fold cross-validation with held-out cells assigned zero weight (mean over three repetitions), shown on a logarithmic scale; the dashed line marks RMSE = 1. Because held-out cells did not contribute directly to the weighted fitting objective, predictions at these cells were unstable and RMSE values greatly exceeded the 0–1 scale of the compositional data. This diagnostic was therefore not used for rank selection.
(TIFF)

S1 Table Transcription errors detected by the internal-consistency audit and corrected after verification against the printed source. Eleven transcription errors were corrected: six cells belonging to the 13 analyzed categories, four values in the printed Total column, and one Other shellfish value outside the 13-category analytical matrix. Corrections to the six analytical cells were incorporated into the catch-composition matrix; corrections to Total and Other shellfish affected only the internal-consistency audit.
(XLSX)

S2 Table Cooperative-years flagged by the internal-consistency audit and outcomes of verification against the printed source. Cooperative-years were flagged when, in the uncorrected transcription, the absolute difference between the printed Total and the sum of the 15 transcribed detail categories was at least 5,000 kg or at least 25% of the printed Total. Forty-five cooperative-years met this criterion. Eighteen were resolved by primary-source verification, whereas 27 remained open and were retained as transcribed.
(XLSX)

S3 Table Recorded weight by category and year, 1962–1972, after correction of verified transcription errors, with lake-wide official totals for comparison. The table reports annual recorded weights for the 13 analyzed categories, the annual sums of the 13 analyzed categories, the 15 transcribed detail categories, and the corrected printed Totals. Lake-wide official total catch and official Ayu catch from the Shiga Prefecture statistical series are included for comparison.
(XLSX)

S4 Table Stability of the *Q* = 5 solution under port-block bootstrap and leave-one-year-, port-, and category-out refitting. The same resampling and exclusion procedures used for the *Q* = 4 reference solution were repeated at *Q* = 5. Values are matched-profile cosine similarities relative to the representative full-data *Q* = 5 solution. The table also identifies the closest *Q* = 4 component for each *Q* = 5 component and summarizes the dominant taxonomic loadings of the *Q* = 5 profiles.
(XLSX)

S5 Table Sensitivity of the *Q* = 4 reference solution to exclusion of the 27 open cooperative-years. The *Q* = 4 model was refitted after excluding the 27 cooperative-years that remained open after source verification (S2 Table; 457 of 484 cooperative-years retained), using the same specification as the full-data analysis. The table reports matched-profile cosine similarities and Spearman rank correlations of normalized component scores relative to the representative full-data solution, together with the dominant taxonomic loadings of the refitted profiles.
(XLSX)
