# Supplementary material for: Does a historical catch record support a stable latent structure in catch composition across ports, taxa, and years? An assessment of cooperative-level catch statistics from Lake Biwa, Japan, 1962–1972
Source: PLoS One. 2026 Oct 14;21(10):e0358946. doi: 10.1371/journal.pone.0358946 (PMC13667830; doi:10.1371/journal.pone.0358946)
Supplement: Supplementary file 1 [file pone.0358946.s001.docx]

**S1 Text. Supplementary methods for data verification, validation, sensitivity analyses, and reproducibility**

**1. Historical-data verification and correction**

The cooperative-level catch data were transcribed from *Fisheries of Lake Biwa*, published in 1973 by the Kinki Regional Agricultural Administration Office and the Shiga Statistical Information Office. The analytical panel comprises 44 fisheries cooperatives observed annually from 1962 through 1972, yielding 484 cooperative-year observations.

Internal consistency was evaluated by comparing the printed Total for each cooperative-year with the sum of the 15 transcribed detail categories: the 13 categories retained for analysis plus Other fish and Other shellfish. The printed Total was not included in the analytical matrix. Cooperative-years were flagged when the absolute difference between the printed Total and the uncorrected sum of the 15 detail categories was at least 5,000 kg or at least 25% of the printed Total.

Forty-five cooperative-years met the flagging criterion. Each was checked against the printed source. Eleven transcription errors were identified and corrected: six analytical cells, four Total values, and one Other shellfish value. Eleven of the flagged cooperative-years contained one or more corrected values; ten of these were classified as RESOLVED, whereas Isoda 1965 remained OPEN because a residual shortfall of 205 kg persisted after correction. Eight additional flagged cooperative-years were confirmed to have been transcribed correctly despite discrepancies internal to the printed source. Thus, 18 flagged cooperative-years were classified as RESOLVED and 27 as OPEN. For OPEN cases, the transcribed values were retained because the source provided no basis for assigning an alternative value. The individual corrections are listed in S1 Table, and all 45 flagged cases and their verification outcomes are listed in S2 Table.

After correction, the 13-category analytical matrix contained a total recorded catch weight of 58,241,377 kg. The matrix contained 6,292 category-by-port-year cells, of which 2,131 (33.9%) were recorded as zero.

**2. NMF fitting and component alignment**

All primary NMF fits were conducted in R 4.4.0 using nmfkc version 0.9.4. Candidate ranks Q = 1–8 were fitted from 30 random starts using random-uniform initialization, convergence tolerance ${10}^{-8}$, and a maximum of 100,000 iterations. Full-data fits used seeds 1–30. For each specification, the fit with the smallest final objective value was retained as the representative solution.

Because component labels are exchangeable across NMF fits, profiles were aligned by maximizing the total cosine similarity between component profiles over all permutations. Component correspondence reported in the manuscript therefore refers to matched taxonomic profiles rather than to the original component numbering returned by individual fits.

**3. Rank diagnostics**

Rank assessment combined reconstruction performance, cross-start stability, consensus diagnostics, predictive validation, and a complementary effective-rank diagnostic. Consensus matrices for Q = 2–8 were constructed from 30 random-initialization fits and summarized using the cophenetic correlation coefficient and the proportion of ambiguous clustering (PAC; ambiguity interval 0.1–0.9). Consensus diagnostics used nmfkc.consensus with nrun = 30 and seed = 123 (the package default, passed explicitly); per-run initialization seeds are generated internally by the package from this value.

The operational reference rank was defined by a study-specific stability rule: all 30 starts had to converge, the minimum matched-profile cosine similarity after rounding to three decimal places had to equal 1.000, and PAC had to be ≤ 0.005. This rule was introduced during revision, after examination of the rank diagnostics, as an operational rule for identifying a reproducible reference solution; it is not intended to estimate a uniquely optimal or true rank.

Repeated bi-cross-validation randomly partitioned both rows and columns into four folds, producing 16 held-out blocks per repetition. Thirty repetitions were conducted using identical partitions across candidate ranks. Repetition $r$ used seed $2000+r$. The smallest rank whose mean held-out RMSE was no greater than the minimum mean RMSE plus one standard error was selected by the one-standard-error rule.

Element-wise cross-validation used 10 folds and three repetitions. Fold assignments in repetition $r$ were generated using seed $5000+r$; the NMF fit for fold $f$ used seed $100r+f$. Held-out cells were assigned zero fitting weight. Because these cells did not contribute directly to the weighted objective, this analysis was treated as a supplementary diagnostic rather than as a rank-selection criterion.

**4. Bootstrap and exclusion analyses**

At Q = 4, stability was evaluated using 500 port-block bootstrap replicates. Ports were sampled with replacement, and all 11 years belonging to each sampled port were retained. The 500 replicates were divided into 10 computational parts of 50 replicates. Part $p$ used seed $30000+p$ for port resampling, and each bootstrap fit used 10 NMF starts with seeds 1–10.

Matched-profile cosine similarities were summarized by their 2.5th, 50th, and 97.5th percentiles and by the proportion of replicate-component matches with cosine similarity ≥ 0.9. These percentiles are perturbation-based stability summaries and are not population confidence intervals.

Leave-one-year-out, leave-one-port-out, and leave-one-category-out analyses used 30 random starts for each refit. In leave-one-category-out analyses, the remaining 12 categories were renormalized to sum to one within each port-year before fitting.

To evaluate dependence on the operational reference rank, the same 500-replicate port-block bootstrap and the same year-, port-, and category-exclusion analyses were repeated at Q = 5. The Q = 5 bootstrap used the same port resamples as Q = 4. Q = 5 results are summarized in S4 Table.

**Exclusion of open cooperative-years.** To assess dependence on the 27 cooperative-years that remained open after source verification, the Q = 4 model was refitted on the 457 retained cooperative-years using the same specification as the full-data analysis (script R/18_open27_sensitivity.R; the exclusion list is derived from verify_against_source.csv, status = OPEN, and matches S2 Table). The refit used 30 random starts with seeds 1–30, as in the full-data fits, and the same convergence settings; consensus diagnostics (cophenetic correlation and PAC) were computed from a separate 30-run consensus analysis (nmfkc.consensus, seed 123). Refitted profiles were matched to the full-data representative solution (results/fit_main_Q4.rds) by taxonomic-profile cosine similarity, and normalized component scores were compared over the retained cooperative-years by Spearman correlation. Outputs are written to results/ (open27_Q4_summary.csv, open27_Q4_component_comparison.csv, open27_Q4_multistart.csv, open27_Q4_profiles_long.csv, open27_excluded_rows.csv, open27_Q4_multifit.rds, fit_open27_Q4.rds, cons_open27_Q4.rds, session_open27_Q4.txt) and summarized in S5 Table.

**5. Sensitivity to data representation and recorded zeros**

Sensitivity to data representation was evaluated by refitting Q = 4 and Q = 5 models to raw catch weights and to log(1 + y)-transformed catch weights. Profiles were matched to the corresponding composition-based solution using cosine similarity.

At Q = 4, the treatment of recorded zeros was additionally varied by assigning zero-valued cells weights of 0.5 or 0 while retaining weight 1 for positive cells. Component profiles were compared with the equal-weight reference fit, and fitted normalized shares at recorded-zero cells were summarized.

**6. Spatial sensitivity and residual spatial structure**

Spatial smoothing was evaluated using the Gaussian-kernel covariate formulation. Port coordinates were projected to JGD2011 / Japan Plane Rectangular CS VI (EPSG:6674). The reference kernel bandwidth was based on the median nearest-neighbor distance of 2.37 km, yielding $\beta_{\mathrm{med}}=0.0890\text{ }\mathrm{km}^{-2}$ and a half-weight distance of 2.79 km. Seven bandwidths spanning $\beta_{\mathrm{med}}2^{-3}$ to $\beta_{\mathrm{med}}2^{3}$ were evaluated. The reference bandwidth used 30 starts, whereas each of the six non-reference bandwidths used 10 starts.

Residual spatial structure was evaluated separately by year using Moran's I with 9,999 within-year permutations. Permutation tests were executed in three blocks of years, with the random seed set to 90000 plus the first year of the block: 91962 (1962–1965), 91966 (1966–1969), and 91970 (1970–1972). The 23 series comprised 13 observed taxonomic shares, four normalized component-score series from the non-spatial model, four from the spatial model, and total-variation reconstruction error from each model. The resulting 253 tests (23 × 11 years) were treated as a single multiple-testing family and adjusted using the Benjamini–Hochberg procedure.

**7. Reproducibility archive**

The reproducibility archive contains the machine-readable transcription, port coordinates, correction and verification ledgers, analysis-ready matrices, R and Python code, the tagged nmfkc source used for fitting, fitted objects, complete validation and sensitivity outputs, publication tables and figures, session information, and file-integrity manifests. Internal filenames retaining “v4” identify the pipeline generation and do not denote the deposited record version.
